# Editorial: Oxidative Damage of RNA: Structure, Function, and Biological Implications - From Nucleotides to Short and Long RNAs in Chemistry and Biology

**DOI:** 10.3389/fmolb.2022.853725

**Published:** 2022-02-25

**Authors:** Lynn Htet Htet Aung, Peifeng Li, Claudia Perez-Cruz, Norbert Polacek, Marino J. E. Resendiz

**Affiliations:** ^1^ Qingdao Municipal Hospital, Qingdao University, Qingdao, China; ^2^ Centro de Investigaciones y Estudios Avanzados, Instituto Politécnico Nacional de México (CINVESTAV), México City, Mexico; ^3^ Department of Chemistry, Biochemistry and Pharmaceutical Sciences, University of Bern, Bern, Switzerland; ^4^ Department of Chemistry, University of Colorado Denver, Denver, CO, United States

**Keywords:** RNA oxidation, oxidation and disease, RNA-protein interactions, oxidative stress, 8-oxoG

## RNA Oxidation

Oxidation of RNA has implications on its structure and function, thus making this an area with great potential for discovery, and of relevance due to the important biological roles involving this biopolymer. Developments in recent years have shown that reactive oxygen species can damage RNA in different cellular compartments, of different types (coding and noncoding), and of various sizes. The present collection of articles represents a compilation of work that highlights the importance of this topic, and the need to further investigate the relevance of oxidative stress and oxidative damage for RNA biology. Understanding how oxidatively damaged RNA is handled and turned-over intracellularly, as well as its biological outcomes, will have an impact on disease prevention/treatment, in addition to the potential discovery of new RNA-based mechanisms. The collection is comprised of reviews, hypotheses, and original research articles. The topics span from the involvement of oxidized RNA in disease progression/development, to mechanistic aspects that involve oxidative stress and oxidatively generated lesions within RNA, to the use of techniques aimed at detecting/characterizing processes involving oxidized RNA. Importantly, it also highlights the interdisciplinary nature of this topic and the potential for collaborations that span medicine, biology, biochemistry, biophysics and chemistry, to mention a few.

In brief, the reviews provide a detailed account on the relationship between RNA oxidation and various diseases; the consequences of oxidatively generated modifications in RNA on protein synthesis and cell signaling; and the impact of oxidative stress in bacteria, specifically RNA machines involved in transcription and translation. Furthermore, a literature supported hypothesis describes the potential processing and decay of oxidized RNA by enzymes with specific roles, e.g., in RNA binding, editing, and degradation; and the role of stress granules in this context. Lastly, the original research articles display the use/development of tools that allow the study of oxidized RNA and related processes, which employ analytical, chemical, and molecular-biology tools to this end.

Fasnacht and Polacek provide a detailed review, and state of research, on oxidative stress in bacteria, while making emphasis that similar/parallel mechanisms are present in other domains of life. The review introduces the reader to various reactive oxygen species (ROS), as well as their sources, while making mention of the potential modifications of various biomolecules/biopolymers, i.e., nucleic acids, amino acids, and cofactors. Then it goes on to discuss how bacteria deal with stress, *via* activation of different stress response regulons, before explicitly describing on the impact of oxidative stress on the central dogma. Beginning with oxidation of DNA, and its role on transcription, where the production of sRNAs, in response to oxidative stress has important implications. The authors then go on to summarize the process of translation by analyzing work carried out on mRNA (elongation/stability), tRNA, as well as the ribosome. In these works, 8-oxo-7,8-dihydroguanine (8-oxoG) is mentioned as the lesion of interest, while RNA modifications such as m^6^A or inosine may play a role in allowing/triggering the degradation of damaged mRNAs. Mistranslation events are also described, along with potential outcomes and handling. Regarding rRNA, the presence of 5-hydroxycytosine (5-OH-C) is mentioned along with the ability of bacteria to use oxidation events in the ribosome functions; where oxidation at select-sites as a function of divalent metal location was elaborated. The authors end with a note on protein oxidation, and the need for further research in this area overall.

On a human health context, Li et al. provide an overview regarding the relationship of oxidized RNA and disease. In this regard, the role of enzymes such as PNPase, YB-1, and PCB 1/2 is discussed, followed by a description of work that relates RNA oxidation with neurodegenerative, cardiovascular, and metabolic diseases, amongst others. Most of the work refers to 8-oxoG as the biomarker for quantifying/detecting/studying oxidative damage on RNA. The authors point out the need to better understand this phenomenon. Delving into a more detailed look on mechanistic aspects, Tanaka and Chock discuss on the impact of oxidative modification on different types of RNA, namely tRNA, mRNA, mtRNA, miRNA, and rRNA. Furthermore, oxidation of RNA and ribonucleotides in signaling pathways is reviewed, e.g., on inflammatory responses, as apoptotic triggers, or in signaling. In this work, abasic sites as well as the nucleobase lesions 8-oxoG, 8-oxoA, 5-OHC, 5-OHU, and 4-pyrimidinone (arising from 2-thiouridine oxidation) are described. The authors make mention that regulatory mechanisms mediated by oxidized RNA remain to be established, and that potential outcomes arising from oxidized RNA could impact cell growth, tumorigenesis, and/or antiviral defense systems. Besides the aforementioned enzymes, cyt c, AUF1, MutT/MTH1, and APE1 (known for its functions on DNA repair) were among other mentioned enzymes with roles involving oxidized RNA. In regards to the intracellular handling of oxidized RNA, Alluri et al., take into consideration that nature has devised ways to eliminate, or repair, such damage/stress on RNA. The authors propose that machinery within stress granules, i.e., ADAR1, Tudor-SN, and STAU1, may play a role in handling the metabolism of oxidized RNA, from RNA binding and editing, to trafficking, to degradation. All in agreement with reports that show accumulation of oxidized RNAs in stress granules. The hypothesized mechanism is promising and should prove useful in providing new research directions in this area.

In the tool development area, Sapio et al. explored the biogenesis of pre-rRNA on mammalian cells, under oxidative stress. To this end, a reporter based on a redox-sensitive green fluorescent protein is described, along with the use of immunofluorescence to detect 8-oxoG in nucleolar RNA. Overall, it was found that oxidative stress leads to perturbations in pre-rRNA transcription and post-transcription processing, although the exact RNA components are yet to be established. Furthermore, the reported protocols and tools should prove useful in measuring the impact of oxidative stress on other types of RNA. On another study, Estevez et al. used mass spectrometry to study the RNA-protective role of proteins, methylation (m^1^G, m^2^G), and hypermodification (5-methylaminomethyl-2-thiouridine, found in bacterial rRNA and tRNA) under oxidative stress. Besides 8-oxoG, the guanidinohydantoin, FapyG (2,6-diamino-4-hydroxy-5-formamidopyrimidine), 8-oxoA, and FapyA lesions were also observed and quantified. Interestingly, the product distribution differed as a function of degree of RNA-protein association and type or ROS generated. The role of amino acids such as tryptophan and lysine as protective entities, given their reduction potentials, is discussed. Lastly, in regards to the decay and processing of mRNA, Phillips et al. explored the processive nature of the exoribonuclease Xrn-1 towards oligonucleotides of RNA containing 8-oxoG. The authors found that this oxidative lesion poses a challenge to Xrn-1 function and results in stalling at these sites. To rationalize this observation, various chemical modifications were introduced (*via* solid-phase synthesis), specifically 8-bromoguanosine, m^1^G, and m^6,6^A, which aided in the final conclusions. The study highlights the potential implications arising from the presence of oxidatively generated lesions in RNA, and the need to further detail mechanisms involved in the handling/decay of oxidized RNA.

Overall, it is worth noting that, while 8-oxoG has been the quintessential oxidative lesion to study oxidation of RNA (and DNA), it is likely that other lesions or oxidatively induced modifications (some mentioned in the various reports herein) may also have important roles. Understanding their impact on RNA structure and function, in its many types, as well as their potential purposes and how they are handled, will be of importance. This is an area of high potential for discovery and we look forward to seeing its implications on various fields and the impact of this compilation on this, and other fields.

